# Toxoflavin analog D43 exerts antiproliferative effects on breast cancer by inducing ROS-mediated apoptosis and DNA damage

**DOI:** 10.1038/s41598-024-53843-1

**Published:** 2024-02-18

**Authors:** Tingyue Wu, Wenjing Liu, Hui Chen, Lei Hou, Wenlong Ren, Longlong Zhang, Jinhui Hu, Haijun Chen, Ceshi Chen

**Affiliations:** 1 School of Life Science, University of Science & Technology of China, Hefei, 230027 Anhui China; 2 Key Laboratory of Animal Models and Human Disease Mechanisms of the Chinese Academy of Sciences, Kunming Institute of Zoology, Chinese Academy of Sciences, Kunming, 650201 China; 3 The Third Affiliated Hospital, Kunming Medical University, Kunming, 650118 China; 4 Key Laboratory of Molecule Synthesis and Function Discovery (Fujian Province University), College of Chemistry, Fuzhou University, Fuzhou, 350116 Fujian China; 5 Department of Breast Disease, Henan Breast Cancer Center, Affiliated Cancer Hospital of Zhengzhou University & Henan Cancer Hospital, Zhengzhou, 450008 China; 6 Academy of Biomedical Engineering, Kunming Medical University, Kunming, 650500 China; 7 The First Hospital of Hunan University of Chinese Medicine, Changsha, 410007 Hunan China

**Keywords:** Toxoflavin, ROS, N-acetylcysteine (NAC), DNA damage, Patient-derived breast cancer organoids (PDO), Cancer, Drug discovery, Oncology

## Abstract

Triple-negative breast cancer (TNBC) is regarded as the deadliest subtype of breast cancer because of its high heterogeneity, aggressiveness, and limited treatment options. Toxoflavin has been reported to possess antitumor activity. In this study, a series of toxoflavin analogs were synthesized, among which D43 displayed a significant dose-dependent inhibitory effect on the proliferation of TNBC cells (MDA-MB-231 and HCC1806). Additionally, D43 inhibited DNA synthesis in TNBC cells, leading to cell cycle arrest at the G2/M phase. Furthermore, D43 consistently promoted intracellular ROS generation, induced DNA damage, and resulted in apoptosis in TNBC cells. These effects could be reversed by N-acetylcysteine. Moreover, D43 significantly inhibited the growth of breast cancer patient-derived organoids and xenografts with a favorable biosafety profile. In conclusion, D43 is a potent anticancer agent that elicits significant antiproliferation, oxidative stress, apoptosis, and DNA damage effects in TNBC cells, and D43 holds promise as a potential candidate for the treatment of TNBC.

## Introduction

Breast cancer has emerged as a predominant malignancy that poses a significant threat to women's health, with its incidence continuing to rise^1^. Breast cancer exhibits significant heterogeneity, manifesting substantial variations both within tumors and among different tumor types, and three major biomarkers known to be differentially expressed in most breast cancer types are estrogen receptor (ERα), progesterone receptor (PR), and human epidermal growth factor receptor 2 (HER2)^2,3^. At present, breast cancer treatment primarily relies on subtype-specific approaches, leading to significant improvements in the overall prognosis of affected patients. However, approximately 20–30% of patients experience recurrence and metastasis within 5 years after surgery, leaving them with limited treatment options upon further progression after multiple lines of therapy^4^. Therefore, there remains an urgent clinical imperative to explore novel treatment strategies. Recently, numerous novel antitumor drugs have emerged for breast cancer treatment. TNBC represents a highly aggressive subtype of breast cancer characterized by the absence of ERα, PR, and HER2 expression in cancerous tissue. This subtype accounts for approximately 15–20% of all breast cancer cases^5,6^. Currently, due to the absence of specific molecular markers, chemotherapy remains the primary first-line adjuvant treatment for TNBC, with a dearth of targeted treatment modalities available^7^. Therefore, it is highly meaningful to discover new TNBC drugs and develop new treatment methods.

Oxidative stress is a prevalent condition observed in tumors and is characterized by an imbalance between oxidation and antioxidation processes within the body. This imbalance leads to a propensity toward oxidation, resulting in abnormal regulation of oxidative signaling and damage to macromolecules^8–10^. Elevated levels of reactive oxygen species (ROS) represent the main factors driving oxidative stress in cells. ROS are byproducts of normal cellular metabolism, primarily resulting from the reduction of oxygen to obtain a single electron, including hydrogen peroxide, superoxide anion, and hydroxyl radical. ROS exhibit high bioactivity, while a series of REDOX reactions within cells contribute to their homeostasis^11^. The mechanism underlying ROS function in cells is intricate^12^. Low levels of ROS serve as crucial second messengers, resulting in various normal cell activities, including cell proliferation, differentiation, and survival. Excessive ROS accumulation can impact lipids, proteins, and nucleic acid macromolecules, leading to altered protein function, lipid peroxidation and damage to DNA and RNA^13^. This accumulation also contributes to the occurrence and progression of various diseases, including tumors. Continued accumulation of ROS beyond a certain threshold induces various biological processes, including cell senescence, apoptosis, and ferroptosis, and ultimately, this plays a crucial role in exerting its antitumor effects^14,15^.

Various drugs that act directly or indirectly on ROS have been employed in successful cancer treatment. The majority of antineoplastic agents used in cancer chemotherapy have the potential to induce significant oxidative stress. Patients treated with these agents exhibited indications of ROS-induced lipid peroxidation in plasma^8^. For instance, taxanes (paclitaxel and docetaxel), vinca alkaloids (vincristine and vinblastine), and antimetabolites (anti-folates) facilitate the release of cytochrome c from the mitochondria. This process triggers cell death and disrupts the electron transport chain, leading to the production of superoxide radicals^16^. Additionally, platinum complexes (e.g., cisplatin, carboplatin, and oxaliplatin) and anthracyclines (e.g., doxorubicin, epirubicin, and daunorubicin) generate exceptionally high levels of ROS^17^. In conclusion, the ability of certain chemotherapeutic agents to disrupt ROS levels presents a therapeutic opportunity for cancer treatment.

Toxoflavin, characterized by the pyrimido [5,4-e][1,2,4] triazine ring, was initially isolated from *Pseudomonas cocovenenans* in 1933^18,19^ and is also obtained from various bacteria, including Burkholderia^20^. It exhibits antibacterial, antiviral, antitumor, and various other useful biological activities, making it a target compound for numerous medicinal chemistry studies^21–24^.

A recent study demonstrated that specific toxoflavin analogs can suppress the overexpression of protein disulfide isomerase (PDI) in glioblastoma and induce the Nrf2 antioxidant response, endoplasmic reticulum (ER) stress response, and autophagy while also acting as novel chemical probes to inhibit PDI as a therapeutic target for brain cancer^25^. In addition, toxoflavin can target the junction between the RNase and kinase domains and inhibit IRE1α by oxidizing the conserved cysteine residues in the active site of IRE1α, inhibiting its activity through oxidation of conserved cysteine residues within its active site. This property enables its application as a small molecule tool to investigate the involvement of IRE1α in ER stress^26^.

Furthermore, toxoflavin exhibits activity on the β-catenin/Tcf signaling pathway, which plays a key role in both normal development and tumorigenesis^27,28^. The application of these compounds in the treatment of other diseases, such as asthma and diabetes, has also been reported^29^. However, there is currently no available information regarding the mechanism by which toxoflavin induces cytotoxicity and cell death specifically in breast cancer cells.

This study involved the synthesis of a series of toxoflavin analogs, followed by the screening of D43 as our lead compound. D43 exhibited continuous upregulation of intracellular ROS, induced DNA damage, and promoted cell death in TNBC, which was reversible upon treatment with NAC. Moreover, patient-derived organoid and xenograft models of breast cancer were employed to demonstrate the significant tumor growth inhibition achieved by D43 while exhibiting favorable biosafety. Consequently, these findings indicated the potential of D43 as a promising drug candidate for treating TNBC.

## Materials and methods

### Cell culture and treatment

The cells used in this study were obtained from the American Type Culture Collection (ATCC, Manassas, Virginia, USA) and were identified by short tandem repeat (STR) analysis. Different cells were cultured with different media, as detailed in Table S1. The medium was supplemented with 10% fetal bovine serum and 1% penicillin/streptomycin at 37 °C with 5% CO_2_ in a humidified incubator.

### Compound synthesis

*O*-Fluorobenzaldehyde (186 mg, 1.5 mmol) was added to a suspension of 255 mg (1.5 mmol) in acetic acid (3 mL) by the one-pot method under stirring at room temperature. The reaction mixture was stirred for 30 min to obtain the intermediate hydrazone. An aqueous solution of saturated sodium nitrite (155 mg, 2.25 mmol) was slowly added to the mixture and stirred for one hour. Next, a water/methanol (v/v = 2 mL/2 mL) saturated solution of sodium dithionite (348 mg, 2 mmol) was added slowly and stirred for one hour. Then, the reaction mixture was diluted with a saturated NaHCO_3_ solution (20 mL), extracted with CH_2_Cl_2_ (60 mL), dried with anhydrous Na_2_SO_4_, and filtered. The crude product was concentrated under reduced pressure. Finally, 2 mL of CH_2_Cl_2_ was added to the solanum flask, and then 20 mL of hexane was added. After ultrasonication and vibration, a large number of yellow solid precipitates were observed, and compound D43 (FZU-0083-085) (206 mg, 48%) was obtained after direct extraction and filtration. The characterization of D43, which is mainly mentioned here, is described in the Supporting Information. Acetylcysteine (HY-B0215, MCE, Shanghai, China) was diluted in DMSO prior to addition to the culture medium.

### Sulforhodamine B assays

Cells were spread in 96-well plates, treated with drugs for a certain period of time, and then fixed with 10% trichloroacetic acid at 4 °C overnight. Afterward, the plates were gently washed and allowed to dry, followed by staining with 100 μL SRB stain (0.4% in 1% acetic acid) for 30 min, and the unbound dye was washed with 1% acetic acid. The plates were allowed to dry, and the stains were dissolved by adding 100 μL Tris buffer (10 mM, pH 10.5). Absorbance was measured at a wavelength of 530 nm using a microplate reader (Infinite M200Pro, Tecan).

### Clonogenic assays

Briefly, 1000 cells were seeded in a 6-well plate and grown overnight. The cells were incubated with different concentrations of D43. The cultures were continued for approximately 2 weeks until sufficient colonies were observed in the control group. The cells were fixed initially with 4% paraformaldehyde and stained with a 0.1% crystal violet solution. Individual colonies larger than 50 cells were counted under a microscope.

### DNA synthesis

An EdU Imaging Kit (HC1010, US Everbright Inc.) was used to measure cell proliferation. In brief, cells were pulse-labeled with 10 μM EdU after drug treatment and cultured for 4 h. They were then fixed in 4% paraformaldehyde at room temperature for 30 min. Glycine (2 mg/mL) was added and incubated for 5 min, followed by washing twice with 3% BSA in PBS. The cells were permeabilized with 0.5% Triton X-100 for 20 min and blocked with 3% BSA. The reaction solution of Click-iT reaction cocktail was added and incubated in the dark for 30 min, followed by incubation with Hoechst33343 solution (1:2000 dilution) for 15 min, and then an anti-fluorescence quencher was added to seal the slides. ImageJ software was used to calculate the proportion of EdU-positive cells after image acquisition.

### Cell cycle analysis

The potential of D43 to regulate the cell cycle was analyzed using propidium iodide (PI) staining. After 48 h of cell starvation, MDA-MB-231 and HCC1806 cells were treated with different concentrations of D43 for 48 h. The cells were collected and fixed with 75% ethanol overnight. Then, 100 μg/mL RNase solution was added, and the cells were stained with PI for 30 min. The DNA content was subsequently analyzed by flow cytometry using a BD LSRFortessa Flow Cytometer.

### Apoptosis analysis

An Annexin Detection Kit (1133534, BD Pharmingen) was used to measure apoptosis. After the cells were treated with a D43 concentration gradient, floating and adherent cells were collected together and centrifuged at 500×*g* for 5 min. The cells were washed with PBS and then stained with FITC/Annexin V and propidium iodide following the manufacturer's instructions. The proportion of apoptotic cells was determined by flow cytometry. The assays were repeated at least three times.

### Western blotting and antibodies

Cells were lysed in RIPA lysis buffer with protease inhibitor. Samples were mixed with 1 × SDS buffer at 98 °C for 10 min, separated by SDS‒PAGE, and transferred to PVDF membranes (Millipore, Germany). The membranes were then blocked with 5% nonfat milk in PBS with 0.1% Tween 20 and incubated with primary antibodies overnight at 4 °C, followed by incubation with horseradish peroxidase-labeled secondary antibodies for 1 h at room temperature. Signals were detected using enhanced chemiluminescence reagent (UE, S6009) by ImageQuant LAS4000 (GE, Germany). Details of the specified antibodies are listed in Table S2.

### Immunofluorescence staining

The cells were seeded on 35 mm glass-bottom petri dishes, treated for a certain period of time, and fixed in 4% paraformaldehyde at 4 °C overnight. They were permeabilized with 0.2% Triton X-100 for 5 min and blocked with PBS containing 3% BSA for 1 h. The fixed cells were washed with PBS and then incubated with a primary anti-phospho-histone H2AX-Ser139 antibody (AP0687, ABclonal) in 3% BSA overnight at 4 °C, followed by incubation with Alexa Fluor 594 (Invitrogen) for 1 h and mounting with mounting medium containing DAPI. Images were taken with a Zeiss fluorescence microscope.

### ROS assays

The level of intracellular ROS was detected using the Reactive Oxygen Species Assay Kit (S0033, Beyotime). The treated cells were digested with trypsin, washed with PBS, and incubated with serum-free medium containing the fluorescent probe DCFH-DA (1:1000 dilution) for 30 min. The samples were shaken every 5 min during this period, and then the cells were washed three times with serum-free medium. The fluorescence intensity was detected by flow cytometry after resuspension in PBS and analyzed using FlowJo software.

### Patient-derived breast cancer organoids

Fresh tumor tissue cut from the patient was placed in precooled DMEM/F12 medium containing 50 μg/mL primary cell antibiotics, the fat and necrotic parts were removed with sterile instruments, washed several times with sterile 1 × PBS, and the tumor tissue was cut into small particles of approximately 1 mm with scissors. The tumor tissues were added to the digestive solution (0.5–1 mg/mL collagenase I, collagenase III, collagenase Iv; 0.05–0.1 mg/mL hyaluronidase; DMEM/F12; 10 mM HEPES; 2% BSA; 0.48 μg/mL hydrocortisone; Y-27632 (5 μM); 50 μg/mL primocin) and digested in a 37 °C water bath for 1–2 h. Until complete digestion, the cells were passed through a 100 μM filter, centrifuged at 500×*g* for 3 min, resuspended in PBS and washed before adding erythrocyte lysate for 3–5 min. After another wash with PBS, the cells were resuspended in Matrigel and seeded in 24-well plates to coagulate for 30 min and ultimately added to the breast cancer organoid medium. When the organoids grew to the required size and number, they were digested and collected, counted after dissolving the matrix gel, and transferred to a 96-well plate. A D43 concentration gradient treatment was administered while PDOs grew to the appropriate size. An ATPase activity kit was used to detect the changes in the viability of TNBC PDO cells treated with different D43 concentrations for 5 days. The study was approved by the Clinical Research Ethics Committee of Yunnan Cancer Hospital (KMMU2023MEC094). Informed consent was obtained from all subjects and/or their legal guardians. The methods were carried out in accordance with the approved guidelines.

### Tumorigenesis

Female nude mice (approximately 5 weeks old), which were obtained from SJA Lab Animal Co. Ltd. (Changsha, China), were injected with HCC1806 cells (1 × 10^6^ cells/spot) by bilateral orthotopic fat pad injection. According to the results of our previous pilot experiment, When the tumor grew to 50 mm^3^, the mice were randomly divided into control and experimental groups (D43 5 mg/kg), and the drug was injected around the tumor every 5 days until the 15th day. Tumor size was measured every 3 days with a Vernier caliper. At the end of the experiment, tumors were harvested for analysis. All mice were kept in specific pathogen-free (SPF) conditions at the Animal Resource Center of Kunming Institute of Zoology, Chinese Academy of Sciences. All animal experiments were conducted in accordance with the guidelines and were approved by the Kunming Institute of Zoology, Chinese Academy of Sciences Animal Care and Use Committee (SMKX-20160305-08). We conducted all animal experiments in accordance with ARRIVE guidelines.

### RNA-seq

HCC1806 cells in logarithmic growth phase were plated in six-well plates and treated with cell compound D43 or DMSO for 24 h. RNA samples were extracted by Trizol method and sent to Lianchuan Bio-Science for high-throughput RNA sequencing. The raw image data file obtained by high-throughput sequencing was converted into the original sequencing sequence after the base calling. fastqc software was used to control the raw data, StringTie software was used to assemble genes or transcripts and FPKM was used for quantification. The R package edage was used to analyze the differential genes between samples. KEGG annotation of differentially expressed genes was performed, and the number of genes corresponding to KEGG annotation was counted and then classified according to biological process, molecular function and cell component. Enrichment analysis was performed on the KEGG annotated genes, and the significant enrichment results were screened according to the adjusted P value < 0.05.

#### Statistics and reproducibility:

All statistical analyses were performed using Prism v.9.0 (GraphPad). The error bars indicate mean ± standard deviation (SD). Two-way ANOVA or two-tailed Student’s t-tests was used to analyze the statistical significance between the control sample and the treated sample. p < 0.05 was considered to be significant.

## Results

### Compound D43 inhibits the growth of TNBC cells

**Figure 1:**
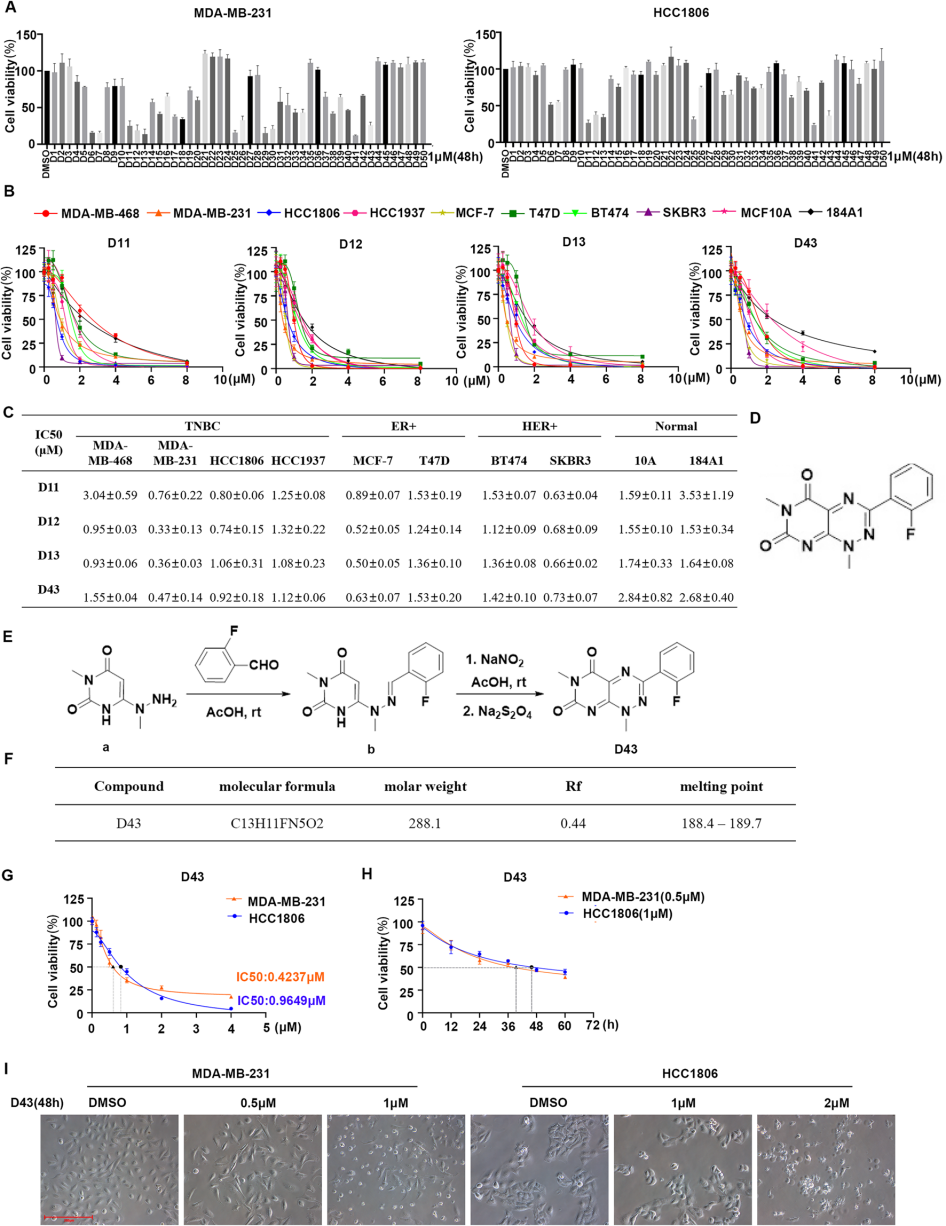
Compound D43 inhibits the growth of TNBC cells. (**A**) SRB experiments were performed to detect the cell viability of HCC1806 and MDA-MB-231 cells after treatment with 50 compounds (48 h, 1 μM). (**B**) IC50 of D11, D12, D13 and D43 in the breast cancer cell lines MDA-MB-468, MDA-MB-231, HCC1806, HCC1937, MCF-7, T47D, BT474, and SKBR3 and the immortalized breast epithelial cell lines MCF10A and 184A1. (**C**) Statistical values of IC50 data. (**D**) The chemical structure of D43. (**E**) The synthesis process of D43. (**F**) The physicochemical properties of D43. (**G**) IC50 of D43 in MDA-MB-231 and HCC1806 breast cancer cell lines. (**H**) The antitumor effect of D43 in MDA-MB-231 (0.5 μM) and HCC1806 (1 μM) cells was detected over time. (**I**) Microscopic morphology of MDA-MB-231 and HCC1806 cells treated with D43.

Cytotoxicity screening is essential to investigate novel anticancer agents during drug discovery. Toxoflavin, a toxin containing a pyrimidine triazine ring, is produced by a variety of bacteria, including Burkholderia spp. Previous reports have demonstrated its antitumor activities. Based on this lead compound, we synthesized a series of related analogs exhibiting drug-like characteristics, including low molecular weight and excellent metabolic stability, to identify potential drugs effective against breast cancer. Initially, the breast cancer cell lines HCC1806 and MDA-MB-231 were chosen, and the cytotoxicity of 50 different analogs of D43 was assessed by the SRB assay. Approximately 10 compounds exhibited less than 50% cell viability in both MDA-MB-231 and HCC1806 cells following a 48 h treatment with 1 μM concentration (Fig. 1A). Based on the properties and structural analysis of the compounds, four compounds, D11, D12, D13 and D43, exhibiting superior solubility were chosen for subsequent experiments. Subsequently, the IC50 values of these four drugs were determined using the SRB assay in normal breast cancer cells and various subtypes of breast cancer cells (Fig. 1B and C). Notably, D11 demonstrated lower efficacy in inducing cell death compared to the other three compounds, whereas D43 showed relatively low sensitivity toward normal breast epithelial cells MCF10A and 184A1 compared to D12 and D13. Although D43 exhibited toxicity to normal cells. We also compared D43 with the lead compound Toxoflavin (Supplementary Fig. 1A and B). Due to its better specific cytotoxic effect on breast cancer cells compared to Toxoflavin, D11, D12, and D13, we have chosen compound D43 for further research. The results indicated that D43 had no specific selectivity for different breast cancer cell lines. Therefore, we chose TNBC which is more aggressive and metastatic and is the type of breast cancer with a worse prognosis. Targeted therapies for TNBC are still in need of further research and development. The specific structure, synthesis process and related physicochemical properties of D43 are shown in Fig. 1D–F.

The IC50 values of D43 were re-evaluated in MDA-MB-231 and HCC1806 breast cancer cells, resulting in values of 0.42 μM and 0.96 μM, respectively (Fig. 1G), and the inhibitory effect of D43 was observed within a time frame of 36–48 h (Fig. 1H). Morphological studies showed that MDA-MB-231 and HCC1806 cells treated with D43 exhibited atrophic, desquamated, and reduced cell numbers. In particular, at high concentrations, the treated cells displayed reduced volume, fragmented nuclei, and numerous small bodies of varying sizes within the cytoplasm (Fig. 1I).

### D43 inhibits DNA synthesis and survival of TNBC cells

**Figure 2:**
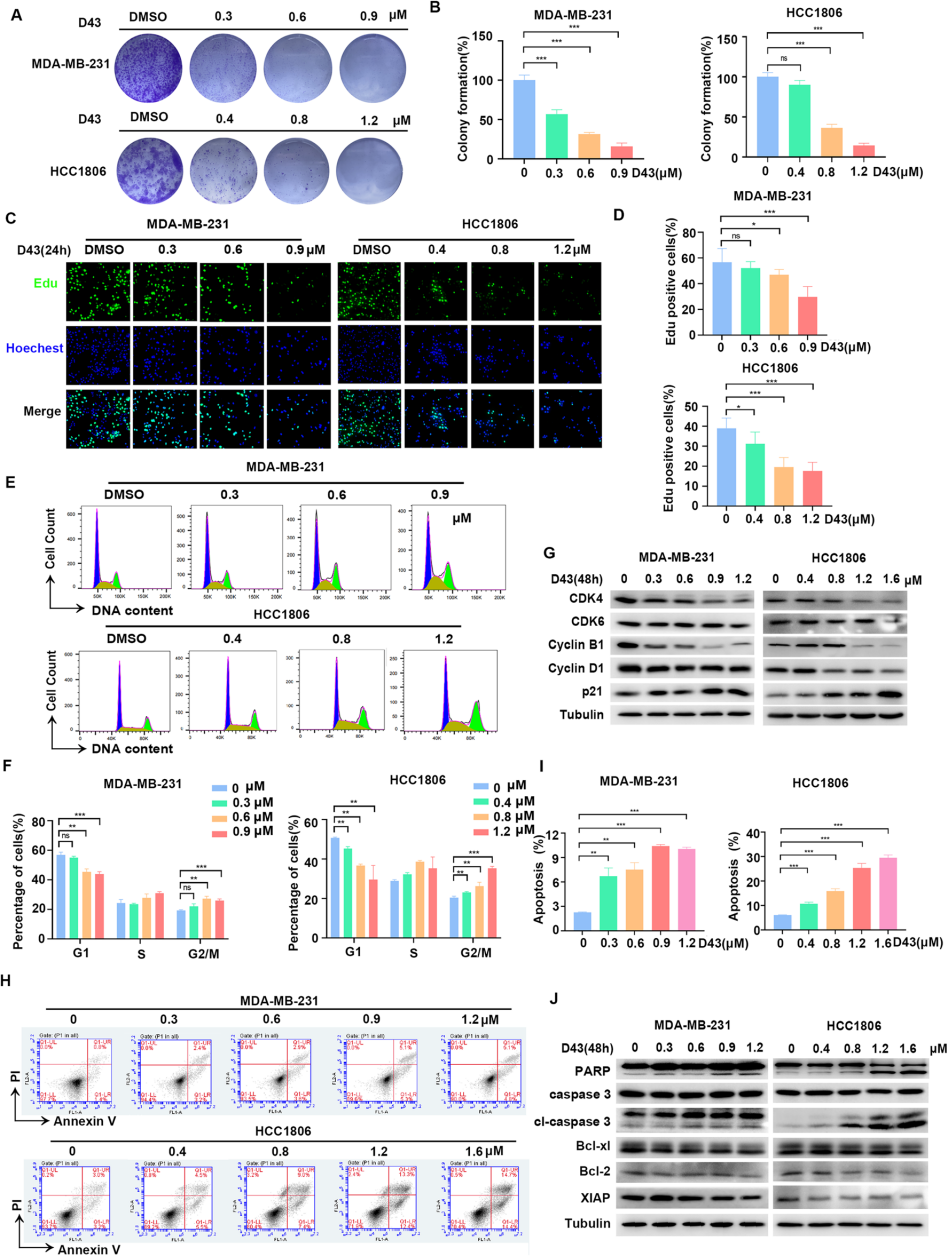
D43 inhibits DNA synthesis and survival of TNBC cells. (**A**) Representative picture of colony formation in MAD-MB-231 and HCC1806 cells incubated with a D43 concentration gradient for 14 days. (**B**) D43 inhibited the colony formation of TNBC cells. Individual colonies were counted under a microscope. (**C**) D43 inhibits the DNA synthesis ability of cells. An EdU probe was used to detect the DNA synthesis ability of MDA-MB-231 and HCC1806 cells treated with different concentrations of D43 for 24 h. Blue represents Hoechst staining, and green represents EdU staining. (**D**) The proportion of EdU-positive cells was counted. (**E**) D43 increased the number of cells in the G2/M phase. Cells were dyed with PI and analyzed with flow cytometry after incubation with D43 (48 h). (**F**) The proportion of each stage of the cell cycle changes is shown. (**G**) D43 regulated cell cycle-related proteins. Western blot images showing the expression levels of cyclin B1, cyclin D1, CDK4, CDK6, and p21 proteins in MDA-MB-231 and HCC1806 cells treated with D43 at different concentrations for 48 h with tubulin as a loading control. (**H**) The proportion of apoptotic subsets was analyzed by flow cytometry after Annexin V-PI double staining with D43 in a concentration gradient treatment for 48 h. (**I**) Statistics showing the proportion of Annexin V-positive cells. (**J**) D43 regulates apoptosis-related proteins. Western blotting was used to detect the expression of apoptosis-related proteins, including PARP, Caspase3 and their splicing forms, and the anti-apoptotic proteins Bcl-xl, Bcl-2 and XIAP were also detected. The samples derive from the same experiment and that blots were processed in parallel. Original blots are presented in Supplementary Material. Data are representative of three independent experiments. *p < 0.05, **p < 0.01 and ***p < 0.001; ns, not significant.

Next, the anticancer activity of D43 was evaluated by assessing its effect on cell growth using the colony formation assay, which demonstrated that D43 exhibited a dose-dependent reduction in the number of single-cell clones compared to the DMSO group (Fig. 2A and B). D43 treatment led to a significant alteration in cell population densities, particularly at low concentrations, in both MDA-MB-231 and HCC1806 cells. Treatment with D43 suppressed DNA synthesis in both cell lines, as indicated by the decrease in the proportion of EdU-positive cells (Fig. 2C and D). Furthermore, treatment with a concentration gradient of D43 for 48 h resulted in a dose-dependent increase in the proportion of cells in the G2/M phase. A notable observation was the substantial arrest of cells in the G2/M phase, leading to a corresponding decrease in the number of cells in the G1 phase (Fig. 2E and F). In line with the flow cytometry results, treatment with D43 significantly suppressed the expression levels of CDK2, CDK6, Cyclin B1, and Cyclin D1 but increased the expression of p21 in MDA-MB-231 and HCC1806 cells, as assessed by Western blot analysis (Fig. 2G).

Then, we investigated the regulation of cell death mechanisms by the pleiotropy of pro- and antiapoptotic proteins using flow cytometry and Western blot analysis. The results showed that D43 treatment significantly induced apoptosis and led to an increased percentage of Annexin V-positive cells (Fig. 2H,I). Subsequent detection of apoptosis-related proteins showed that D43 treatment, compared to DMSO treatment, promoted the dose-dependent cleavage of Caspase 3 and PARP (Fig. 2J). Additionally, D43 treatment decreased the expression levels of the antiapoptotic proteins Bcl-xl, Bcl-2, and XIAP. These findings suggest that D43 may induce apoptosis by activating the mitochondria-dependent apoptotic pathway.

### D43 induces DNA damage by increasing the level of ROS in TNBC cells

**Figure 3:**
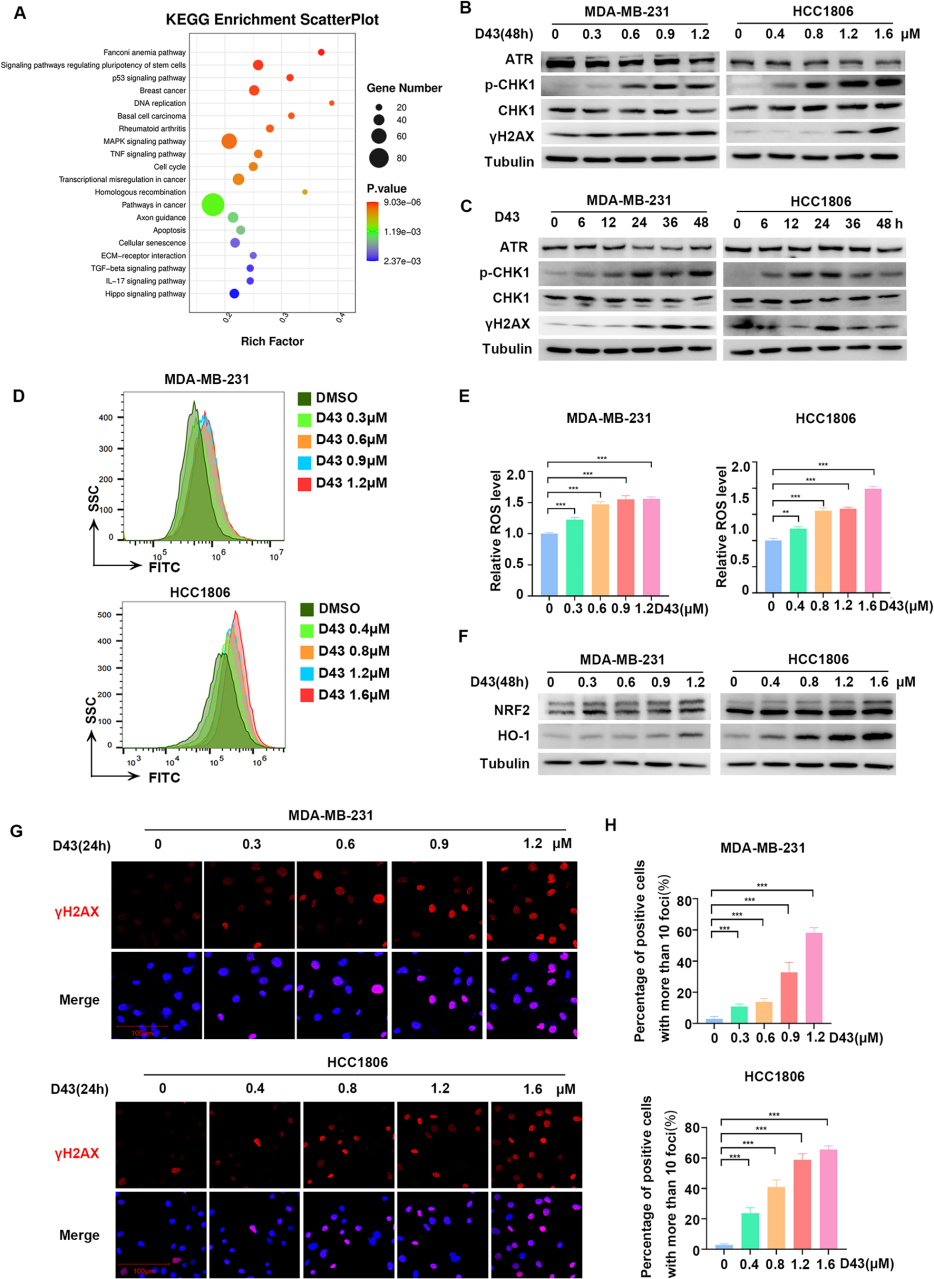
D43 induces DNA damage by increasing the level of ROS in TNBC cells. (**A**) RNA sequencing of HCC1806 cells treated with D43 (1 μM) for 24 h revealed a KEGG enrichment scatterplot. (**B**) D43 caused DNA damage in a concentration-dependent manner. Western blot assays were performed to detect the expression of ATR, p-CHK1, CHK1, and γH2AX in MDA-MB-231 and HCC1806 cells treated with D43 at different concentrations for 48 h with tubulin as a loading control. (**C**) D43 caused DNA damage in a time-dependent manner. After treatment of D43 with a time gradient (0, 6, 12, 24, 36, 48 h), the above proteins were also detected by Western blotting. The samples derive from the same experiment and that blots were processed in parallel. Original blots are presented in Supplementary Material. (**D**) D43 stably increased the ROS level in TNBC cells. MDA-MB-231 and HCC1806 cells treated with a D43 concentration gradient for 48 h were labeled with the fluorescent probe DCFH-DA, and ROS levels were detected by flow cytometry. (**E**) Statistics of relative ROS levels. (**F**) D43 was able to activate the NRF2-HO-1 signaling axis in response to oxidative stress in a dose-dependent manner. Western blot assays were performed to detect the expression of NRF2 and HO-1 after treatment with D43 at different concentrations for 48 h. (**G**) D43 treatment resulted in a higher number of γ-H2AX foci. MDA-MB-231 and HCC1806 cells were treated with a D43 concentration gradient for 24 h, and the expression of γ-H2AX was detected by immunofluorescence staining and recorded by high-resolution fluorescence microscopy. (**H**) Four samples were taken from each treatment group to count the proportion of positive cells with more than 10 foci in the cells. Data are representative of three independent experiments. *p < 0.05, **p < 0.01 and ***p < 0.001; ns, not significant.

Subsequently, we investigated the molecular mechanisms by which D43 induces cell cycle arrest and apoptosis in TNBC cell lines. HCC1806 cells were subjected to transcriptome sequencing after treatment with either DMSO or D43 for 24 h to identify potentially enriched gene sets and signaling pathways that could shed light on the underlying mechanism of D43 (Fig. 3A). KEGG analysis revealed enrichment of numerous genes and signaling pathways related to DNA replication and damage, such as the “Fanconi anemia pathway”, “DNA replication” and “homologous recombination”, as well as significant regulation of metabolism-related genes. Consistently, treatment with D43 resulted in the downregulation of ATR expression, which is an apical kinase of DNA damage and replication stress, upregulation of CHK1 phosphorylation, and increased expression of γ-H2AX in a concentration- and time-dependent manner (Fig. 3B and C).

ROS are well known to cause DNA damage. To test whether D43 generates ROS, we measured ROS levels in MDA-MB-231 and HCC1806 cells treated with D43 concentration gradients by flow cytometry, and the results showed a stable increase of ROS levels with significant differences (Fig. 3D and E), providing additional evidence that D43 potentially triggers the mitochondria-dependent apoptotic pathway in TNBC cells. D43 was also able to activate the NRF2-HO-1 signaling axis in response to oxidative stress in a dose-dependent manner (Fig. 3F). To investigate the impact of D43 on the response of TNBC cells to DNA double-strand breaks, γ-H2AX immunofluorescence staining was employed to analyze the number of γ-H2AX foci in MDA-MB-231 and HCC1806 cells treated with D43, revealing that D43 treatment resulted in a higher number of γ-H2AX foci (Fig. 3G and H). Based on these results, we inferred that D43 may induce DNA damage by increasing ROS levels.

### NAC rescued D43-induced DNA damage and ROS upregulation

**Figure 4:**
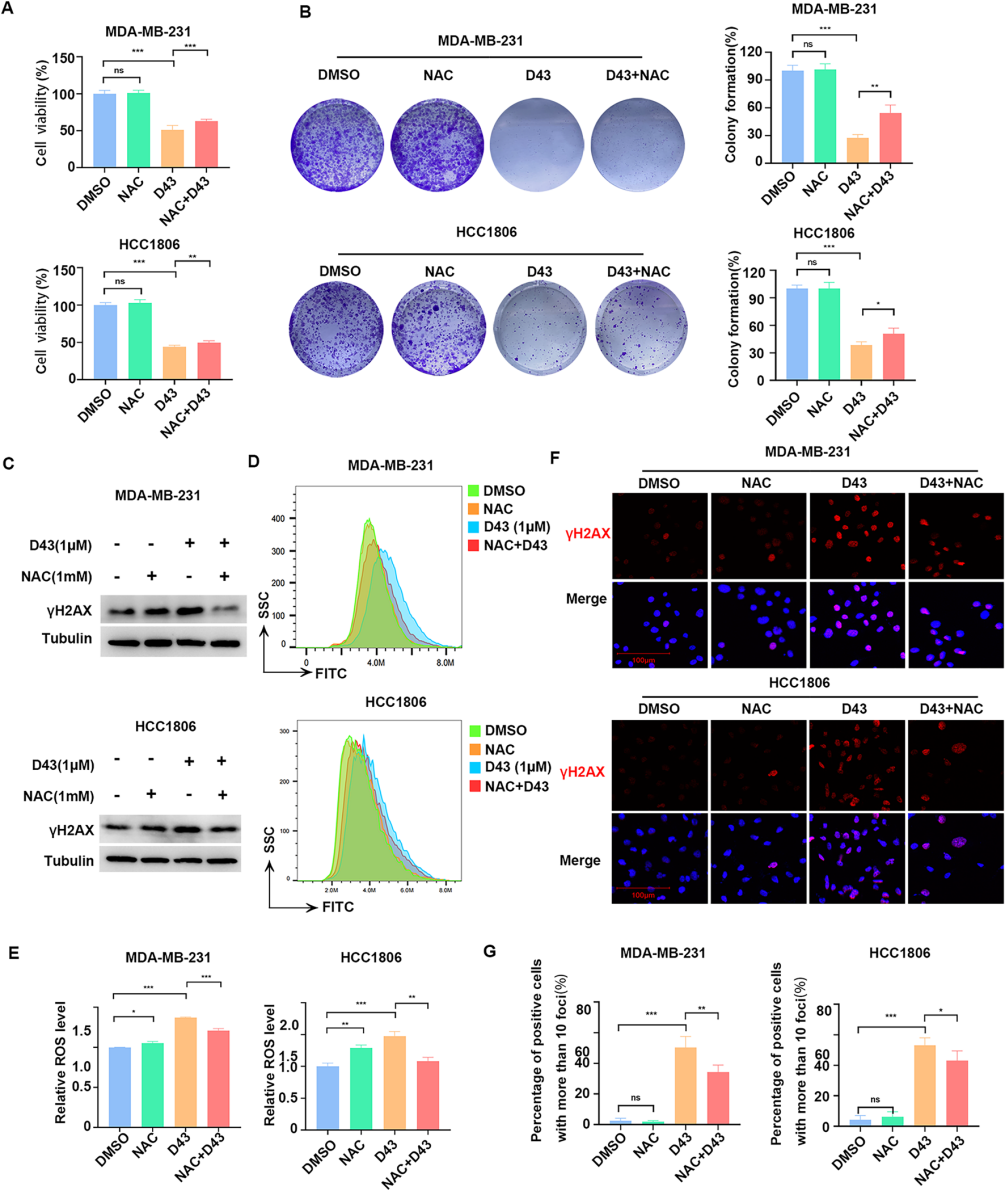
NAC rescued D43-induced DNA damage and ROS upregulation. (**A**) NAC partially alleviated the cell death induced by D43 in HCC1806 and MDA-MB-231 cells. After pretreatment with NAC (1 mM) for 6 h, MDA-MB-231 (0.5 μM) and HCC1806 (1 μM) cells were treated with D43 for 48 h, and cell viability was verified by SRB. (**B**) Cell proliferation was verified by colony formation assay after treatment with NAC and D43, respectively, or in combination. (**C**) Western blot analysis showed that pretreatment with NAC (1 mM, 6 h) antagonized D43-induced increase of γH2AX protein levels. The samples derive from the same experiment and that blots were processed in parallel. Original blots are presented in Supplementary Material. (**D**) NAC effectively antagonized the D43-induced upregulation of ROS. MDA-MB-231 and HCC1806 cells were treated with D43 or NAC for 48 h and labeled with the fluorescent probe DCFH-DA, and ROS levels were measured by flow cytometry. (**E**) Statistics of relative ROS levels. (**F**) NAC antagonized the upregulation of γ-H2AX foci formation induced by D43. After NAC pretreatment for 6 h, MDA-MB-231 (0.5 μM) and HCC1806 (1 μM) cells were treated with D43 for 24 h, and the expression of γ-H2AX was detected by immunofluorescence staining and recorded by high-resolution fluorescence microscopy. (**G**) Four samples were taken from each treatment group to count the proportion of positive cells with more than 10 foci in the cells. Data are representative of three independent experiments. *p < 0.05, **p < 0.01 and ***p < 0.001; ns, not significant.

Several studies have demonstrated that oxidative stress can induce apoptosis^30,31^. Under normal physiological conditions, a delicate equilibrium exists between oxidative and antioxidant mechanisms within cells. However, in various pathological states, excessive production of oxygen free radicals occurs, disrupting this equilibrium and resulting in oxidative stress. There is evidence that oxygen free radical scavengers such as NAC, superoxide dismutase (SOD), vitamin E, and glutathione peroxidase can effectively inhibit oxidative stress-induced apoptosis^32^. The results obtained from SRB and colony formation experiments demonstrated that NAC partially alleviated the cell death induced by D43 in HCC1806 and MDA-MB-231 cells (Fig. 4A and B). Furthermore, our observations revealed that NAC effectively antagonized the D43-induced increase of γH2AX protein levels and ROS levels (Fig. 4C–E) and the formation of γ-H2AX foci (Fig. 4F and G) in HCC1806 and MDA-MB-231 cells. In conclusion, the above experiments provided additional evidence that D43 is capable of inducing DNA damage by increasing ROS levels, and this damage can be partially rescued by the antioxidant NAC.

### D43 inhibits the growth of patient-derived TNBC organoids and xenograft tumors

Breast cancer is characterized by tumor cell heterogeneity and variations in the tumor microenvironment, leading to diverse patient characteristics. To assess the therapeutic effects of D43 on TNBC, we utilized the PDO model, which originates directly from patient tumor tissue. This three-dimensional culture method effectively preserves cancer stem cells and faithfully reproduces the cellular diversity and heterogeneity observed in the primary tumor while maintaining its histological and genetic characteristics^33–35^.

**Figure 5:**
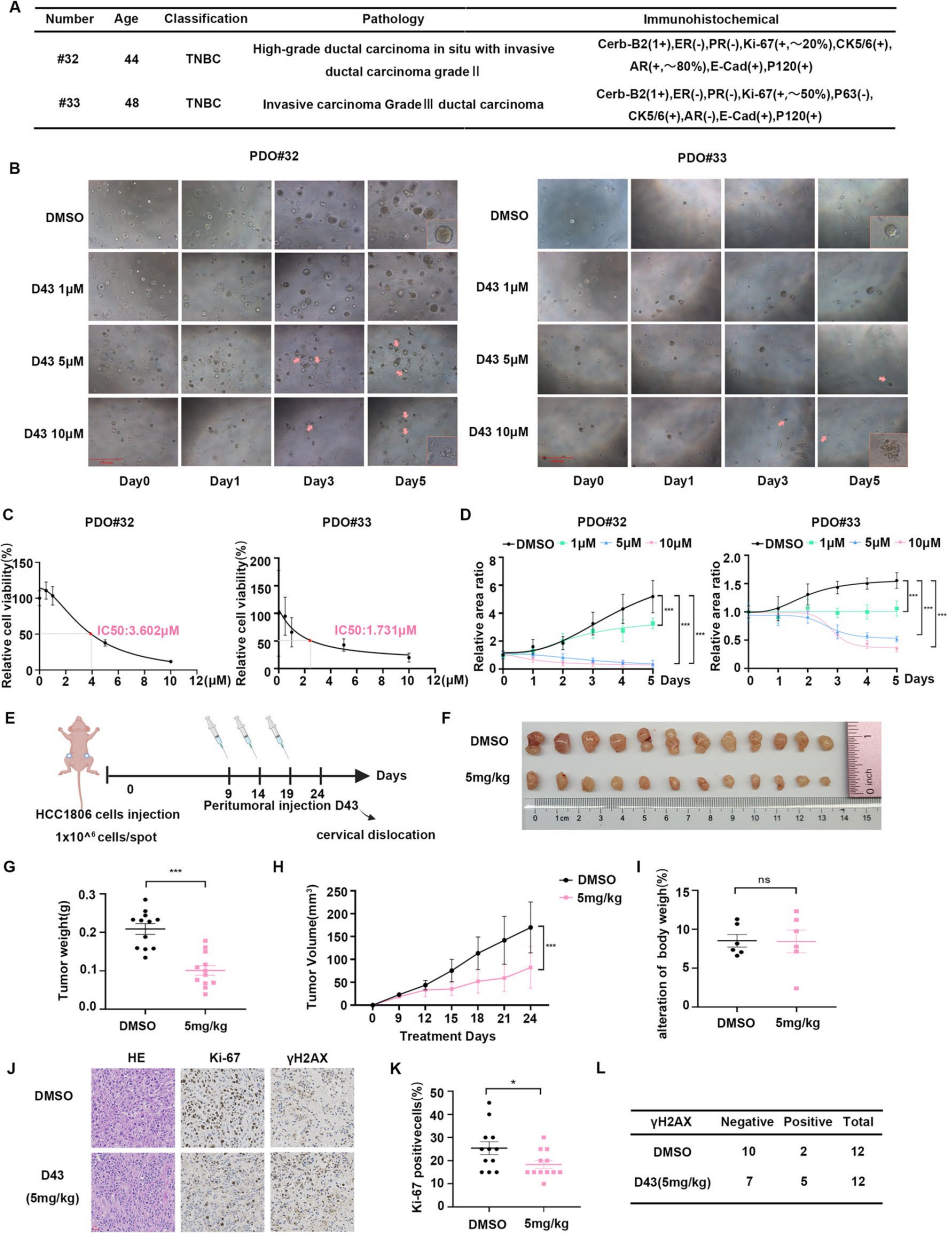
D43 inhibits the growth of patient-derived TNBC organoids and xenograft tumors. (**A**) Tissue information of TNBC patients sampled from Yunnan Cancer Hospital. (**B**) Treatment with D43 compromised the structural integrity of TNBC PDOs. Morphological records of breast tumor organoids at different D43 treatment concentrations for different days. The red arrow points to the disintegrating or completely disintegrating TNBC PDOs. (**C**) D43 inhibited the ATP activity of TNBC PDOs. An ATPase activity kit was used to detect the changes in the viability of TNBC PDO cells treated with different D43 concentrations for 5 days. (**D**) D43 had a significant inhibitory effect on the growth of TNBC PDOs. The area of PDOs was calculated by ImageJ, and the growth curve was drawn. (**E**) Schematic diagram of subcutaneous tumor model construction in nude mice and administration time and frequency. (**F**) D43 significantly suppressed tumor growth in nude mice. After 15 days of continuous administration, the mice were sacrificed by cervical dislocation, and tumors were removed and photographed. (**G**) The tumor weights of control (DMSO) and experimental (5 mg/kg) mice are shown. (**H**) The tumor volume was weighed every 3 days after the beginning of the drug administration treatment and is shown as a line chart. (**I**) Changes in body weight of mice before and after D43 administration. (**J**–**L**) Immunohistochemistry was used to detect the expression of Ki-67 and γH2AX in tumor tissues, and γH2AX immunoreactivity was scored semiquantitatively. *p < 0.05, **p < 0.01 and ***p < 0.001; ns, not significant.

Using two patient-derived tumor tissues diagnosed as TNBC subtypes, we created PDO models by isolating and digesting the tumor tissues, followed by their combination with Matrigel (Fig. 5A). The cells were subcultured and treated with different concentrations of D43. The results demonstrated that D43 compromised the structural integrity of TNBC PDOs. The PDOs, which initially displayed distinct edges and complete morphology, disintegrated into single cells or cell populations upon D43 treatment, leading to the loss of their original structure (Fig. 5B). Additionally, ATPase activity assays performed on the fifth day of D43 treatment revealed its ability to inhibit cell viability in TNBC PDOs (Fig. 5C). Evaluation of IC50 values indicated differences between organoid models derived from different patients (#32 PDO: approximately 3.6 μM; #33 PDO: approximately 1.7 μM), highlighting variations in the response to D43 among these models. In addition, we employed ImageJ software to perform fitting calculations and statistical analyses on the PDO area, confirming the significant inhibitory effect of D43 on TNBC PDO growth (Fig. 5D).

The in vivo impact of D43 on xenograft tumors formed by HCC1806 was assessed using a subcutaneous tumor formation model in immunodeficient mice (Fig. 5E). The results revealed a significant reduction in tumor weight and volume in the group administered D43 compared to the control group (Fig. 5F–H). Furthermore, D43 had minimal impact on the body weight of nude mice (Fig. 5I), and it did not affect the levels of serum aminotransferase (ALT/AST) and creatinine (Cr) (Supplementary Fig. 2A–C). These findings indicate that D43 exhibited no liver or kidney toxicity, indicating its favorable biological safety in vivo.

Moreover, immunohistochemistry analysis of tumor specimens demonstrated that D43 significantly decreased the presence of Ki-67-positive cells in the tumor tissues. Additionally, semiquantitative scoring of γH2AX immunoreactivity revealed a higher percentage of positive tumors in the D43-administered group (Fig. 5J–L).

## Discussion

TNBC is a pathological classification associated with a poor prognosis after breast cancer treatment due to its high heterogeneity and aggressive nature^36^. Previous studies have described the antitumor properties of toxoflavin in various tumor cells^23,24^. The toxicity of toxoflavin is presumed to originate from inhibition of the respiratory chain^19^. Toxoflavin derivatives were identified as active substances in several high-throughput screening campaigns^25,37–39^. However, the mechanisms underlying toxoflavin's cytotoxicity and cell death in breast cancer remain largely unknown. Therefore, this study aims to investigate the safety of toxoflavin, its specificity in targeting breast cancer cells, its effect on cell cycle processes, and the underlying mechanisms of apoptosis.

Conducting a dose‒response analysis is crucial in the development of chemotherapy drugs. This study analyzed the cytotoxic effects of compounds on breast cancer cells, and we observed that D43 demonstrated a potent anticancer effect in TNBC. D43 effectively inhibited cancer cell survival and DNA synthesis, leading to cell cycle arrest at the G2/M phase. Furthermore, D43 downregulated the expression levels of cyclin-promoting proteins. Additionally, D43 induced apoptosis by activating the caspase cascade and inhibiting the expression of anti-apoptotic proteins. Mechanistically, we discovered that D43 induces cellular damage and death by activating ROS signaling. Finally, we demonstrated the significant inhibitory effects of D43 on TNBC utilizing both patient-derived organoid and xenograft tumor models, highlighting its excellent efficacy and favorable biological safety profile.

A notable observation in our research is that RNA-seq analysis of enriched gene sets or signaling pathways in breast cancer cells treated with D43 revealed a substantial number of genes associated with DNA replication and damage. Previous works have emphasized the importance of ROS in tumor research. ROS are capable of affecting biological macromolecules, causing DNA damage and promoting the activation of oncogenes. However, excessive levels of ROS can cause DNA damage. ROS oxidize diphosphatidylglycerol anchored to cytochrome C in mitochondria and release cytochrome C^40^. Moreover, ROS present in the cytoplasm can induce the release of cytochrome C, leading to cell apoptosis in a Bax-dependent manner through the activation of the JNK and p38 MAPK signaling pathways^41^. In addition, ROS can also promote tumor cell ferroptosis^42,43^ or senescence^44,45^. Hence, ROS play a dual role as both a facilitator and a detriment in the context of tumors.

Another noteworthy finding in our study is that D43 significantly downregulates the expression of DNA damage repair-related genes, suggesting that D43 may impair the self-repair ability of cancer cells while intensifying the effect of ROS. The inhibition of crucial signaling molecules involved in DNA damage repair systems, such as ATM, ATR, CHK1, CHK2, and essential DNA damage repair enzymes, can effectively counteract ROS. This inhibition impedes DNA damage repair, resulting in an augmented accumulation of gene mutations and subsequent genome instability^46,47^.

Chemotherapy and radiotherapy are reliant on their cytotoxic effects on DNA, inducing irreparable genome-wide damage in proliferating cancer cells with defective DNA repair pathways. This damage ultimately leads to apoptosis. Therefore, chemotherapy and radiotherapy remain the primary treatments for numerous unresectable or metastatic malignancies. Currently, clinical chemotherapy drugs targeting DNA damage in breast cancer predominantly consist of anthracyclines, platinum, and PARP inhibitors, among others^48–50^. However, due to cancer cells' remarkable ability to resist and adapt to anticancer drugs, dysregulation of DNA damage repair (DDR) can lead to either genotoxic hypersensitivity or drug resistance^51^. Combining drugs such as immunotherapy with those that directly or indirectly synergistically block the DDR pathway is one of the strategies to overcome drug resistance. The search for novel drugs for TNBC aims to enhance treatment sensitivity.

Although D43 has been shown to induce cell death by accumulating ROS and increasing DNA damage, the specific direct targets of D43 compounds remain elusive. Exploring the specific targets could yield valuable insights into the underlying mechanisms that drive the anticancer effects of D43, thereby facilitating the development of highly targeted therapeutic approaches.

Organoids are complex 3D structures that closely resemble the structure and function of in vivo organs, effectively reproducing the histological features of the original tumor^52^. Compared with traditional 2D cultured tumor cell lines and PDX models, PDOs have the advantages of individualization, a short culture cycle, and small differences from the source tissue. These organoids can be used for rapid high-throughput drug screening to determine drug sensitivity results and predict patient responses to novel treatment regimens. By combining chemotherapy with targeted agents or multiple targeted agents during a patient's initial treatment, truly personalized tumor therapy can be achieved^53^. Organoid culture is a valuable platform for studying breast cancer risk, drug development, mechanisms, conducting drug screening, and developing individualized treatments specifically for breast cancer^54,55^. Additionally, employing organoid models for in vitro susceptibility evaluation of D43 enhances the realism of drug testing, thereby improving the reliability and reproducibility of research findings.

In conclusion, our study highlights the remarkable anticancer properties of D43, a novel compound, both in vitro and in vivo. D43 exhibits significant antiproliferative effects, induces apoptosis, and causes DNA damage in TNBC cells. These effects are likely attributed to its continuous activation of oxidative stress and disruption of cellular homeostasis. Notably, D43 demonstrates selectivity for both normal breast epithelial cells and breast cancer cells, suggesting its potential as an ATR expression inhibitor and DNA damage inducer in clinical applications. Combining D43 with other DNA damaging agents or PARP inhibitors may enhance therapeutic efficacy, warranting further investigation. Overall, our research sheds light on the innovative properties of D43, including its molecular mechanism of action, potential as a targeted therapy, efficacy in organoid models, and possible use in combination therapy. These findings contribute to the advancement of treatment options for TNBC and pave the way for further exploration in this field. Future investigations should focus on validating these results and exploring potential clinical applications of D43, offering new directions for improving the management of TNBC patients.

### Supplementary Information

Supplementary Information.

## Data Availability

All data generated or analysed during this study are included in this published article and its supplementary information files.

## Abbreviations

ATR: Ataxia telangiectasia and Rad3-related protein
Bcl-2: B-cell lymphoma 2
Bcl-xl: B-cell lymphoma-extra large
Chk1: Checkpointkinase 1
Cdks: Cyclin-dependent kinases
DDR: DNA damage response
FBS: Fetal bovine serum
IC50: Half maximal inhibitory concentration
HO-1: Heme oxygenase-1
IRE1α: Inositol-requiring enzyme 1
NAC: N-acetylcysteine
Nrf2: Nuclear factor erythroid 2-related factor 2
PDI: Protein disulfide isomerase
PI: Propidium iodide
REDOX: Reduction–oxidation reaction
ROS: Reactive oxygen species
XIAP: X-linked inhibitor of apoptosis protein
